# The Future of Exercise-Based Cardiac Rehabilitation for Patients With Heart Failure

**DOI:** 10.3389/fcvm.2021.709898

**Published:** 2021-08-04

**Authors:** Andrea Passantino, Laura Adelaide Dalla Vecchia, Ugo Corrà, Simonetta Scalvini, Massimo Pistono, Maurizio Bussotti, Fabiana Isabella Gambarin, Domenico Scrutinio, Maria Teresa La Rovere

**Affiliations:** ^1^Istituti Clinici Scientifici Maugeri Spa SB, IRCCS Division of Cardiac Rehabilitation, Bari, Italy; ^2^Istituti Clinici Scientifici Maugeri Spa SB, IRCCS Division of Cardiac Rehabilitation, Milan, Italy; ^3^Istituti Clinici Scientifici Maugeri Spa SB, IRCCS Division of Cardiac Rehabilitation, Veruno, Italy; ^4^Istituti Clinici Scientifici Maugeri Spa SB, IRCCS, Division of Cardiac Rehabilitation, Lumezzane, Italy; ^5^Istituti Clinici Scientifici Maugeri Spa SB, Division of Cardiac Rehabilitation, Montescano, Italy

**Keywords:** heart failure, cardiac rehabilitation, exercise, telerehabilitation, cied recipients, heart failure with preserved ejection fraction

## Abstract

Cardiac rehabilitation (CR) is a comprehensive program that includes exercise training, titration of medical therapy, lifestyle modification, educational support, and psychosocial assessment. All these components are safe and beneficial resulting in significant improvements in quality of life, functional capacity, mortality, and hospital readmission. Current guidelines support its use in a broad spectrum of cardiac disease. This review focuses on exercise-based CR for heart failure (HF) patients in whom CR is a recommended treatment. Exercise should be prescribed according to a personalized approach, optimizing, and tailoring the rehabilitative program to the patient's characteristics. Specific CR programs are dedicated to older patients, those with HF and preserved ejection fraction, and recipients of cardiac implantable electronic devices or left ventricular assistance device. Telemedicine may increase CR participation and overcome some of the barriers that limit its utilization.

## Introduction

Heart failure (HF) is a major public health issue. The prevalence of HF is rapidly growing, due to increasing incidence, aging population, and improvements in the treatment of acute cardiovascular diseases. Patients affected by chronic HF (CHF) have impaired quality of life (QoL) and high risk of recurrent hospitalizations. Mortality of advanced HF is higher than that of breast or colon cancer (1).

Shortness of breath and fatigue are frequent and disabling symptoms that limit work capacity and social life, while QoL becomes very poor in the advanced stages of the disease.

Cardiac rehabilitation (CR) is a comprehensive intervention that has been proven effective in improving functional capacity and QoL in CHF patients.

The core components of CR are exercise training (ET), optimization of medical therapy, counseling, education, and psychological support (2).

Notwithstanding a vast amount of medical literature, there are some gaps in our knowledge in this field, and many issues remain to be addressed.

Even now, there is large room to ameliorate functional outcome by tailoring exercise prescription and by choosing the best setting for CR, according to the patient's characteristics. A rational choice of the best setting where to implement rehabilitative procedures, which could optimize resource allocation and improve patients' adherence to CR; about this issue, the use of telemedicine is very appealing.

Lastly, the role of CR in special populations, such as subjects with HF with preserved ejection fraction (HFpEF), older frail patients, and cardiac implantable electronic devices (CIEDs) or left ventricular (LV) assist device (LVAD) recipients, needs an in-depth evaluation.

## Cardiac Rehabilitation and Heart Failure: A Historical Perspective

Until the early 1950's, patients with myocardial infarction were advised to observe many weeks of bed rest; thus, they developed severe muscle deconditioning.

In the successive decade, early mobilization (EM) for coronary patients (i.e., “the chair therapy”) (3) was implemented. In the meanwhile, some pivotal studies, first of all those of Jeremey Morris, began to demonstrate the benefit of exercise in preventing cardiovascular disease in the general populations (4). The beneficial effects of physical activity were later observed in patients with coronary heart disease, demonstrating how a structured program of aerobic ET could reduce mortality by about 20% in people who survived a myocardial infarction (5).

Nowadays, aerobic ET is considered a life-saving therapy for patients with coronary heart disease; most guidelines recommend CR after myocardial infarction to reduce mortality and morbidity. Over time, also patients after cardiac surgery became eligible candidates for CR.

Extension of CR programs to HF patients is more recent; several years were necessary to demonstrate that physical training may be safe and beneficial in this population. Until the 1970's, ET was contraindicated for HF patients with dilated cardiomyopathy (6, 7). Safety concerns magnified the low propensity to exercise HF patients, because of an increased susceptibility to arrhythmias and hemodynamic instability.

However, in the early 1980's, some researchers started to train patients with HF, obtaining a significant and positive effect from long-term training. The beneficial effect of training was mainly ascribed to peripheral adaptations (8). In studies, there was no evidence of increased risk during rehabilitation sessions. Summarizing these first experiences, in 1990, Coats asserted “… home-based physical training programs are feasible even in severe chronic heart failure and have a beneficial effect on exercise tolerance, peak oxygen consumption, and symptoms. The commonly held belief that rest is the mainstay of treatment of chronic heart failure should no longer be accepted” (9).

The physiological background of physical training in HF patients was the “muscular hypothesis” (10). According to this theory, abnormal skeletal muscles are a central derangement in CHF: low cardiac output leads to metabolic adaptations and consequent wasting of skeletal muscle; ergo-reflex activation causes reflex sympathetic activation, vasoconstriction, and excessive ventilatory response to exercise. Therefore, patients complain of increased dyspnea and fatigue during exercise and limit their physical effort. The consequent physical inactivity further worsens muscle abnormalities in a vicious circle.

The relevance of peripheral abnormalities in HF patients was confirmed by the evidence that maximum consumption of oxygen (VO_2peak_) obtained during an incremental cardiopulmonary exercise test (CPET) is a powerful predictor of prognosis in HF (11).

Mechanistic studies demonstrated the beneficial effect of exercise on some relevant pathophysiological targets. In HF patients, exercise reduces peripheral markers of inflammation and neuroendocrine activation, corrects endothelial dysfunction, and has beneficial effects on mitochondrial ultrastructure and fiber type distribution in skeletal muscle (12–15). Furthermore, ET results in a partial reversal of chronotropic incompetence, a common finding among patients with HF (16). Although, the mechanisms responsible for impaired chronotropic responsiveness are likely multifactorial, recent data have indicated that reduced sinus node beta-receptor responsiveness and not intrinsic sinus node dysfunction plays a role (17).

Some issues and possible pitfalls may complicate the accomplishment of clinical trials related to CR effects. For example, it is unethical to create a control group that will not receive any rehabilitation treatment; the weight of the different components of a CR program (namely, exercise, education, medical advice, and psychological support) is not consistent among studies; the prescription of modality and intensity of exercise may differ substantially among protocols.

The effect of CR could change according to the setting in which rehabilitation takes place (in-hospital (InH), ambulatory, outpatient services, home, supervised, and not supervised) and according to the duration of training. Many small, single-center trials demonstrated the benefits of CR in terms of improved exercise capacity in HF patients. Functional capacity has been evaluated by different ways: by questionnaires, the 6-min walking test (6MWT), or measuring VO_2peak_ during a CPET, with the latter being the gold standard.

A meta-regression analysis reported by Uddin (18) evaluated the predictors of exercise capacity after exercise-based CR in patients with coronary heart disease and HF. Sixty-one trials were included in the study: 35 were based on exercise only programs. The average number of sessions per week was 3.6, with a mean duration of 41 min. Trials differ in the modalities of exercise prescription with regard to duration, frequency, and intensity.

To overcome differences in reporting of functional outcomes, authors reported the difference in functional capacity at follow-up by standardized mean differences. A significant improvement in functional capacity was found across all trials (0.95 standard deviation unit). The difference of VO_2peak_, when reported (in 41 trials), between control and intervention, was 3.3 ml/kg/min.

Uddin et al. concluded that exercise-based CR improved significantly exercise capacity; the magnitude of improvement was similar in CHF and coronary heart disease patients.

Taylor et al. confirmed the benefit of ET on QoL and functional capacity by an individual participant data (IPD) meta-analysis. Thirteen trials including a total 3,990 patients, 97% of whom had HF with reduced EF (HFrEF), entered the analysis. After a 12-month follow-up, patients who were trained showed a significant increase of distance walked at the 6MWT (mean 21 m) and of QoL, measured by the Minnesota Living With Heart Failure test (MLWHFT) (19).

While the evidence that CR is able to improve functional capacity and QoL is largely accepted, its effects on hard clinical end-points, like death or hospitalizations, are more controversial.

In 2004, the Exercise training Meta-analysis of trial in Chronic heart failure (Extramatch) collaborative group reported a reduction in all-cause mortality (hazard ratio (HR) 0.65, 95% CI 0.46–0.92) and in the composite end-point of mortality and hospital admission (HR 0.72, 95% CI 0.56–0.93) (20). These results derived from a meta-analysis including nine trials and 801 patients.

In 2009, the results of HF-Action, an US National Institute of Health funded trial, were reported (21). HF-Action is, to date, the largest controlled clinical trial aimed to test the efficacy and safety of ET among patients with HF. The study enrolled 2,331 outpatients with New York Heart Association (NYHA) II to IV HF and LVEF of 35% or less. Patients in the exercise group first participated in a structured, supervised exercise program that included walking, treadmill, or stationary cycling and then patients began a home-based ET.

The primary end-point was a composite of all-cause mortality or all-cause hospitalization. ET resulted in a not-significant reduction in the primary end-point (HR 0.93, 95% CI 0.84–1.02).

After some baseline characteristics were adjusted, a statistical reduction of the incidence of the primary end-point was observed. The overall interpretation of these results was that exercise had a modest, if null, effect on clinical end-points.

Interestingly, the improvement in functional capacity evaluated by mean of the 6MWT and the CPET was quite low and not clinically relevant, even if statistically significant.

After 3 months of follow-up, patients in the exercise group showed a median 20-m increase at the 6MWT compared with a 5-m increase in the control group, while the increase of VO_2peak_ was 0.6 ml/kg/min in the exercise group compared with 0.2 ml/kg/min in the control group.

At 12 months, the increase in VO_2peak_ was significantly higher in the exercise group (0.7 vs. 0.1), while there were no differences in the distance walked during the 6MWT.

However, this trial confirmed the safety of the ET program.

Adherence to exercise program was a main issue of the trial: at 12 months, only 30% of patients in the active arm of HF-Action were adherent.

Interesting findings about the optimal intensity of exercise were reported by a subsequent analysis of the data from HF-Action, in which there was a reverse J-shaped association between exercise volume and clinical risk. Patients who sustained a moderate workload regimen, 3 to <5 metabolic equivalent (MET)-h, and 5 to <7 MET-h per week had a significant reduction in cardiovascular mortality and hospitalizations (22).

In a further secondary analysis of HF-Action trial, a modest increase in VO_2peak_ over 3 months was associated with a lower composite rate of all-cause mortality and all-cause hospitalization. Every 6% increase in VO_2peak_ was associated with a 5% lower risk of the primary end-point (HR 0.95; CI 0.93–0.98; *p* < 0.001) (23).

However, the reliability of exercise capacity as a surrogate end-point for major clinical events has been questioned in a meta-analysis of randomized clinical trials, showing a low level of association between improvement of exercise capacity and mortality/hospitalization (24).

An IPD meta-analysis, the ExTraMATCH II collaboration, was published in 2018. The analysis, which included individual data from 18 studies and 3,912 patients, concluded that exercise-based CR does not have a significant effect on the risk of mortality and hospitalization in HFrEF (25).

Similar results were found in meta-analysis that explored the Cochrane Central Register of Controlled Trials and that concluded that CR has no impact on mortality but that it may reduce the risk of all-cause hospital admissions and HF-related hospital admissions (26).

These conflicting results confirm the inherent difficulty in designing and performing trials exploring the benefit of exercise-based CR (27, 28).

### Predicting Individual Responses to Exercise Interventions: Role of Cardiopulmonary Exercise Test and Echocardiography in Guiding Exercise Prescription

Prescription of exercise may be complex. Recently, the Paracelsus concept of dose-related toxic effects of drugs has been extended to ET (29, 30), in particular when dealing with CHF patients (31), and implies the need to establish the “dose” of exercise needed to achieve the benefits rather than even harmful effects. Therefore, debate exists on the level of exercise recommended in the presence of cardiovascular risk and/or diagnosed cardiovascular disease at different ages and in CR (32–43).

An exercise prescription is a recommended physical activity program designed in a systematic manner in terms of frequency, intensity, time, type, volume, and progression. The sum of these domains is known as the FITT-VP6 principle (33).

Such prescription should be preceded by an exhaustive clinical assessment, including risk stratification, transthoracic echocardiography (TTE), and exercise testing (CPET if available). It should take into account the fitness level of the patient, his/her individual preferences, the presence of comorbidities and any potential contraindication to physical exercise, and the current therapy. In fact, the success of a rehabilitation plan, which includes ET, is based on adherence to the plan itself, which can be obtained more easily through a personalized prescription, just like the usual prescription method for drugs, meeting the patient's preferences, and focusing on his/her awareness and motivation.

Moreover, a complete TTE examination and a maximal exercise testing (CPET if available) are preliminary tools aimed at identifying any specific contraindications to the ET programs recommended by all the major European and American cardiovascular associations in the current guidelines for CR (35–43).

In addition, to define the degree of monitoring and supervision required during training and, ultimately, to set up a tailoring prescription, a basal TTE is the main test to assess the degree of structural heart disease, in particular systolic and diastolic dysfunction for both ventricles, valvular disease, and pulmonary artery hypertension, before starting a CR program (44).

It is useful to monitor the CHF patient during the rehabilitation and to describe its beneficial effects in the short and long term (45).

A maximal incremental exercise test (if CPET is not available) is useful to prescribe exercise intensity, which is the most important element in obtaining gains while maintaining the safety of the cardiovascular rehabilitation program. Maximal incremental exercise also allows to identify any potentially unsafe electrocardiographic abnormalities and to stratify risks in HF patients (46–50).

During a maximal exercise test, it is necessary to monitor heart rate (HR) dynamics, in order to obtain resting HR value (HR_rest_), maximal HR at peak exercise (HR_max_), and HR reserve (HR_max_ – HR_rest_) (HRR) (33). Nevertheless, a CPET remains the gold standard for maximum aerobic exercise intensity prescription (34).

CPET measures ventilation, O_2_ consumption (VO_2_), and CO_2_ production (VCO_2_) and provides objective measures of respiratory, metabolic, and cardiovascular responses throughout a progressive maximal exercise. Specifically, it is possible to identify the first anaerobic threshold (AT), to which the gradually increasing anaerobic metabolism is added to the aerobic metabolism with the production of lactic acid, which is however, buffered by the bicarbonate system, and a second threshold, the respiratory compensation (RC) point, beyond which the pH begins to decrease resulting in additional stimulation of ventilation.

CPET can precisely furnish the HR and the work rate (WR) related to the metabolic transition points [HR at AT and RC (HR_AT_ and HR_RC_) and WR at AT and RC (WR_AT_ and WR_RC_)].

Aerobic ET for patients with heart disease has traditionally been carried out using a constant WR method even though it is true that any physical activity can be useful for training an HF patient, such as dancing, walking, or practicing yoga, with the aim to increase the patient's adherence (32).

A standard training session includes a warm-up (10 to 15 min), an endurance training (15 to 30 min), and a cool-down (3 to 6 min). The American College of Sports Medicine (ACSM) classifies exercise in six classes according to VO_2_ measured at the peak of a maximal exercise (VO_2peak_): very light (<25% of VO_2peak_), light (25–44%), moderate (45–59%), heavy (60–84%), very heavy (>85%), and maximal (100%) (43).

Remarkably, the metabolic and gas exchange kinetics during a constant WR exercise not only differs from a linearly increasing WR but also depends on exercise intensity (32).

Actually, it is possible to identify four exercise intensity domains: light to moderate (< AT), moderate to high (>AT but < RC), high to severe (>RC), and severe to extreme [near WR reached at peak exercise (WR_MAX_)]. The physiological characteristics of the four exercise intensity domains are summarized in Table 1.

**Table 1:** Exercise intensity domains.

	**AT**		**RC**	
	**Light to**	**High to**	**Moderate to**	**Severe to**
	**moderate**	**severe**	**high**	**extreme**
Borg scale	9–12	13–16	>16	18–20
% VO_2peak_	45–55	55–80	>80	~100
HR reserve %	HR_MAX_ 60–70	70–90	>90	~100
Blood lactate (mmol/l)	<2.0	2.0–4.0	>4.0	
Exercise duration	>30 min	20–30 min	3–20 min	<3 min
Training modality	Continuous	Continuous	Interval	Interval

*AT, anaerobic threshold; RC, respiratory compensation point; HR, heart rate*.

The type of exercise intensity domain will determine the use of a maximum physical capacity percentage, e.g., a percentage of the VO_2peak_ or of the HR_max_.

Alternatively, HR-based methods can be cautiously used when the determination of AT and RC is not possible, keeping in mind that there will always be a share of patients training below AT or above RC.

In sports medicine, in order to obtain the best result, the training load should be as high as possible, avoiding both exceeding the second threshold and an exaggerated sympathetic activation in CHF patients (30).

In HF patients, the choice of the ideal ET intensity is even harder to establish.

A great variety of aerobic exercise intensities (from < AT to near WR_MAX_) has been tested, and nearly all domains have been proven to be effective in improving HF patients' exercise tolerance. The ability to induce reverse LV remodeling and improvements in LVEF, on the contrary, has been demonstrated only for moderate-to-high-intensity and high-to-severe-intensity aerobic training (50).

The >AT but < RC is usually preferred affording a better safety/efficiency balance, despite the lack of unanimous consensus in the literature (32, 50).

Numerous studies favor high-intensity training and interval training (51–53), i.e., those in which the patient exercises beyond the RC point; however, these protocols are rarely used in clinical practice being associated with some discomfort and a poor patient compliance (32, 41–54).

Depending on the chosen training intensity, it is possible to identify target HR training zone (THR) through the Karvonen formula: THR = [(HR_MAX_ – HR_REST_) × % Intensity] + HR_REST_ (54).

Currently available guidelines suggest for cardiac patients a THR into a range from 60 to 80% of the HRR, similar to the relative HR of 40–80% of VO_2peak_ or 50–90% of HR_MAX_ (31–42).

The major advantage of adopting HR as the variable for exercise prescription and monitoring is its linear relationship to both VO_2_ and WR during incremental exercise (55). Therefore, after assessment of the HR_MAX_, the intensity of ET can be identified as the percentage of this value corresponding to a specific percentage of VO_2peak_ (54).

This relation becomes even closer in healthy people, when examining the reserve HR percentage compared with the VO_2_ reserve percentage (55).

%HRR has been identified to equal percentages of VO_2_ reserve (VO_2pea_k – VO_2REST_) in both normal individuals performing either treadmill or cycle exercise and in cardiac patients (55).

The ACSM has adopted %HRR as the gold standard for exercise intensity indirect evaluation (43).

In HF patients, such relationship seems to be true only for those who have a resting HR of 50 to 60 beats per minute (bpm) and taking a full dose of beta-blockers (56).

Nonetheless, the most accurate method for aerobic physical ET for HF patients who take beta-blockers remains the training within the range between HR_AT_ and HR_RC_ (57).

Depending on whether or not on beta-blockers, patients should receive different recommendations; those who are not on beta-blockers should exercise at HR of 70% of the HRR or at 85% of HR_MAX_, while those on beta-blocker therapy should reach a target HR of 60% of the HRR or 80% of HR_MAX_ (57).

The greatest limit of Karvonen method is the issue related to calculate HR_AT_ ± 10% in a high percentage of HF patients. Considering these data, HR_AT_ identified during maximal CPET remains the best method to prescribe ET intensity in HF (58).

These recommendations do not apply instead in patients with chronotropic incompetence or atrial fibrillation, and in some patients with a pacemaker; in these cases, exercise prescriptions should consider WR_MAX_ or VO_2peak_ (58, 59).

The recommendations are clearer regarding the volume of training that should be achieved; indeed, it should reach an expenditure of 1,500 kcal/week (43).

In the first phase of the rehabilitation plan, especially after a recent episode of congestive HF, it is usually suggested to start with a low intensity of exercise. Therefore, it is necessary to work on the domains of both frequency and duration of each exercise session, rather than intensity. Intensity should be maintained quite constantly, close to the lowest limit of the moderate-intensity range, as suggested by the Borg and rating of perceived exertion (RPE) scales (40–50% VO_2peak_, 50–60% HR_max_, 40–55% HRR, and 2.5–3.0 or 11–12).

Thereafter, training frequency usually remains quite constant, whereas, changes in training intensity and duration are progressively implemented in order to increase the weekly training stimulus.

If a preliminary test cannot be performed, all the information about training range should be approximated by theoretical formulas considering the patient's age.

Simpler tests such as the 6MWT can be used in setting up aerobic training. Patients are instructed to walk as far as possible in a 6-min time period, taking rest periods if necessary. The 6MWT is performed on a flat 30- to 50-m-long corridor while collecting physiological parameters including HR, blood pressure, blood oxygen saturation, and the perceived exertion. The total distance walked in meters is the main outcome of the test. The effort tolerance data can be used to personalize exercise programs by focusing on an improvement in aerobic performance (60).

Borg scales, that is, the original “Category Scale” (RPE Borg scale), which rates exercise intensity from 6 to 20, and the “Category-Ratio Scale” (CR10 Borg scale), which uses a numerical range from 0 to 10, allow the implementation of a further functional, easy, and inexpensive method of physical training monitoring in HF through a subjective RPE. An RPE Borg scale rate of 9–12 is equivalent to a light-to-moderate exercise remaining under AT. An RPE range of 13–16 (“somewhat hard” to “hard”) corresponds to an exercise included between the AT and RC and is slightly associated with a 70–90% range of HR_MAX_ and a 50–85% range of VO_2peak_ (59).

Finally, CPET and TTE may represent a helpful combination for training prescription, evaluation of treatment efficacy, and outcome prediction (61). If CPET is somehow underused, the combination to TTE is even rare. However, an undoubted role has been demonstrated in HF patients, in particular in those with diastolic dysfunction and preserved systolic function (62, 63).

## Home-Based Rehabilitation: The Emerging Role of Telemedicine

Following 2016 European Society of Cardiology (ESC) guidelines (64), “cardiac rehabilitation (CR) must be integrated in the overall provision for patients with HF.” However, the optimal choice of setting (InH, ambulatory, home, etc.,) in which to perform CR remains debated.

As previously reported by some of us (65), there is a positive impact of an InH-CR program on all-cause mortality, as well as on hospital readmissions in patients with HF. Clinical benefits and cost-effectiveness of CR in patients with HF are already well-described; nevertheless, the participation remains low. In our study (65) that involved >140,000 incident HF cases admitted to hospitals in the Lombardy Region of Italy between 2005 and 2012, less than a third of patients were referred to InH-CR following decompensation episodes occurring in their CHF course. InH-CR was associated with a 43% decrease in the risk of mortality and 31% decrease in the risk of readmissions. In the UK, <20% of patients discharged from hospital after a diagnosis of HF are referred for CR, and in those who do have a referral, there is a 6% better mortality outcome at 12 months (66). For this reason, and to overcome some of the barriers (including geographic and logistical), new possible settings have been studied. There is a need for alternatives to conventional, supervised, center-based rehabilitation including CR options supported by telemedicine. Affordable home-based programs can make CR more available and should be included as part of the CR offer, especially as these are supported by evidence of cost-effectiveness. The explosion in new technologies, advancements in telemedicine, the presence of remote patient monitoring, and patient-generated data *via* mobile health together with smartphone application have the potential to create a safe, effective, and standardized home-based setting as an alternative to InH and ambulatory setting (67).

Hybrid programs that are facility-based combined with home-based models, telephone- and Internet-based models, and telemedicine CR are rapidly expanding, especially in rural areas with no physical access or during the recent coronavirus disease 2019 (COVID-19) pandemic.

COVID-19 led to the increased use of CR in CHF patients; with successful existing home-based CR programs serving as a basis, advancements in telemedicine and remote monitoring devices have the potential to create a safe, effective, and standardized home-based program to enrich the different settings in which CHF patients could afford CR (68).

The use of technology can improve patient awareness and engagement. Patient and caregiver experience can allow close monitoring of patients who may be geographically disadvantaged or with barriers. These efforts, coupled with wearable technologies, global positioning devices, and activity monitoring, can help to monitor physical activity and progresses, reduce recidivism to inactivity, and maintain the benefits achieved. Our previous studies demonstrated the efficacy of telerehabilitation in CHF patients with important comorbidities, such as chronic obstructive pulmonary disease (COPD) (69) and in older CHF patients (70).

Three major findings emerged from these studies: first, most patients completed the CR program satisfactorily using the integrated telerehabilitation platform, and the participation rate remained above 90%. Second, no serious adverse cardiovascular events occurred throughout the study period. Finally, exercise tolerance significantly improved upon completion of the CR program, as assessed by the 6MWT. Although, older patients are widely considered unfamiliar with the use of electronic devices, such as smartphones or tablets, our results indicated that home-based CR using a real-time remote system could be used in older persons. Home-based CR model can provide patient-centered management; however, the participants need to be educated and encouraged to adhere to the instructions of clinicians and training specialists. As home-based CR has the advantages of accessibility and cost-effectiveness, it is promising that well-organized protocols can be applied in this home-based setting. A recent systematic review demonstrated that home-based CR seems to be similarly effective for improving clinical outcomes and QoL in patients with coronary artery disease or HF (71). A simplified home-based CR program could be tailored to the patient's needs, paying attention to his/her limitations and to the living environment, incorporating activities, such as brisk walking or jogging for cardiovascular exercise and calisthenics or elastic bands for strength training. It is also important to assess whether this type of program produces exercise-related improvements in participants similar to those seen with a more comprehensive CR program that include the use of specialized exercise equipment (e.g., an elliptical trainer, exercise bicycle, or similar equipment for cardiovascular ET; elastic bands or hand or machine weights for strength training).

The explosion in new technologies should be tailored to make home-based program safe and effective. Moreover, further, long-term studies are still needed to provide solid evidence. This process will require testing these new technologies within CR and creating evidence-based guidelines for home-based CR program. It will be important to assess the impact of different models on uptake and adherence of the different programs, to assess the impact of digital enhancement to delivery of home-based CR, to explore model to address multi-morbidity, and to capture the health utility of therapies for HF. The evolution toward hybrid model (both InH and home-based) could be the next step for a successful future for the outcome of CHF patients.

## Special Populations

Although, CR programs were originally addressed to younger patients with HFrEF, more recently, there has been a major increase in the number of patients with HFpEF, often older and frail subjects, and recipients of cardiac devices. Future CR programs should be designed for these populations, which could benefit from a comprehensive rehabilitation program, tailored to their clinical characteristics.

### Heart Failure With Preserved Ejection Fraction

HFpEF represents a significant percentage of HF population; this phenotype is common in older individuals, women, and hypertensive patients.

HFpEF has poor prognosis and impaired QoL; furthermore, no medical treatment has proven effective in improving its clinical outcome.

In HFpEF, cardiac, vascular, and muscular abnormalities cause dyspnea and exercise intolerance (72).

Impaired LV relaxation during exercise leads to increase in pulmonary capillary wedge, pulmonary artery pressures, and LV filling pressure; the atrial contribution to diastolic function, that, owing to LV chamber stiffens, is fundamental and is often lost because of the frequent occurrence of atrial fibrillation. Chronotropic incompetence is another pathophysiological derangement involved in reduced exercise capacity.

Impaired LV–arterial coupling is a mechanism of the reduced exercise tolerance in HFpEF patients, which has demonstrated a blunted increase in muscle arterial blood flow during exercise. Endothelial dysfunction, microcirculation abnormalities, and arterial stiffness are other possible causes of vascular abnormality.

Anatomical and functional abnormalities of skeletal muscles are important contributors to impaired aerobic capacity. Reduced O_2_ extraction, increased intermuscular adipose tissue, altered mitochondrial density and biogenesis, reduced type I (oxidative) fibers, with a lower type I/type II fiber ratio, reduced mitochondrial content, and oxidative enzyme activity are consistent with a lower maximal oxidative capacity (72).

Against this pathophysiological background, ET, an intervention that targets the above-mentioned derangements, could improve exercise capacity and effort tolerance in HFpEF. Few small, randomized clinical trials examined the effects of endurance ET in HFpEF patients with inconsistent results.

The training protocols used in most studies consisted of a combination of supervised InH and home-based outpatient programs, including aerobic exercise, endurance, and resistance training, walking, and treadmill and bicycle ergometer. Most of the protocols ranged 12–16 weeks, with a frequency of two to three sessions weekly, lasting 20–60 min per session.

Most single studies and meta-analysis found a significant improvement of functional capacity, measured by VO_2peak_ and 6MWT. On the other hand, the effect on QoL and TTE is controversial (73).

Fukuta et al. published a meta-analysis including a total of eight trials and 436 HFpEF patients. They concluded that ET improves VO_2peak_, 6MWT, and QoL. On the contrary, no effect was demonstrated on LV function and structure (74).

In agreement with this conclusion, it is generally thought that the improvement of VO_2peak_ is primarily secondary to non-cardiac, peripheral adaptations (75, 76).

Kamiya et al. reported a multicenter retrospective cohort study performed in 15 hospitals. Of the 3,277 patients hospitalized for acute HF (AHF), 26% (862) participated in an outpatient CR program. Of the 3,277 patients, 26% (862) participated in outpatient CR. After propensity matching, HRs associated with CR participation were 0.77 (95% CI, 0.65–0.92) for composite outcome, 0.67 (95% CI, 0.51–0.87) for all-cause mortality, and 0.82 (95% CI, 0.67–0.99) for HF-related re-hospitalization. Interestingly, in HFpEF, CR participation was associated with a more favorable prognosis (77).

In conclusion, there is low-quality evidence that exercise could improve functional capacity in HFpEF. There is no evidence of effect on hard end-points, like overall survival and hospitalizations.

Further, studies on homogeneous HFpEF populations are necessary to confirm the effects of ET.

### Older and Frail Patients

The prevalence of HF increases in the last decades of life. Owing to the current socio-demographic, epidemiological, and health trends that are contributing globally to a continuous, constant, and rapid increase in the number of elderly people, the prevalence of elderly people HF is expected to further increase in the next decades. In older patients, physiological reduction of physical performance, due to aging process, is aggravated by HF-related functional decrements.

In the older people, relevant comorbidities (orthopedic, pulmonary, neurological, and sarcopenia) may cause a further worsening of functional capacity. In these patients, atrial fibrillation is a common findings; the loss of sino-atrial function leads to further worsening of exercise tolerance and physical performance.

When the impairment of multiple physiological systems, along with a deficit in cognitive and social domains, goes so far as to make patients more vulnerable to stressors, the patients become frail.

Most exercise trials in CHF have enrolled younger, middle-aged people; the mean age of patients enrolled in the exercise arm of HF-Action trial was 59.3 years. The study included nearly 500 patients, age >70 years; they had relatively low grade of comorbidity and were not comparable with very old and frail people.

A meta-analysis that included studies on older patients demonstrated a positive effect of physical training on QoL and aerobic capacity regardless of the ET protocols (78).

In older people without frailty, benefits of aerobic exercise are comparable with those observed in younger patients.

Some evidences exist that frail HF patients participating in CR may have meaningful reduction of frailty (79); some studies report a 20–40% improvement in the frailty score in participants in general CR programs (80, 81).

The Rehab study (82) explored the effect of a rehabilitative intervention in 349 older patients (mean age 72.7 ± 8 years, 52% women, 53% with HFpEF) hospitalized for acute decompensated HF. Most of patients were frail or prefrail. They were randomized to receive a tailored, multidomain physical rehabilitation intervention that started in the hospital and continued for 12 weeks post discharge. Exercise was prescribed according to disability measured in four domains: balance, mobility, strength, and endurance.

The primary outcome of the study was the score on the short physical performance battery (SPPB) after 3 months. The SPPB is a measure of global physical function, validated in older and frail people.

The secondary outcome was the rate of re-hospitalization for any cause at 6 months.

After 3 months, patients in the intervention group showed a significantly higher score on the SPPB than did the control group. All the three components of the SPPB improved in the intervention group. Substantial benefits of the intervention were observed in the 6-min walking test and in QoL, measured by the Kansas City Cardiomyopathy Questionnaire (KCCQ). No significant differences were observed in the rate of the secondary outcome.

These results underscore the relevance of an early, tailored rehabilitative intervention in older people, hospitalized for AHF, in order to improve their physical function and QoL. The potential benefits of CR in older people are great. The multidimensional nature of a rehabilitative intervention provides opportunities to address most of the relevant clinical and functional issues of older and frail patients, improving the quality of health management. In older people improving QoL, reducing symptoms and mood disorders is the most relevant outcome to pursue.

The preliminary evaluation of older and frail persons should not be limited to VO_2peak_ determination (when possible) or to the result of the 6MWT. It should include specific tests to measure frailty, physical performance, and cognitive impairment. CR programs should be designed according to patient's characteristic including a tailored combination of rehabilitative procedures like aerobic exercise, gait training, balance training, and resistance training.

### Recipients of Cardiac Implantable Electronic Device

Implantable cardioverter defibrillators (ICDs) and cardiac resynchronization therapy (CRT) are recommended therapies for selected patients with HF. In the HF-ACTION trial, 23% of patients in the active group had an ICD (21).

During the CR program, some specific issues need to be addressed.

In CIED recipients, comprehensive CR offers advantages beyond the ET-driven improvement of functional capacity. It is an opportunity to test and optimize the setting of stimulation device (rate responsiveness, atrioventricular (AV) synchrony, AV delay, and synchronization in CRT) and to offer a psycho-social intervention, aimed to improve clinical and mental status of CIED recipients, reassuring them about physical activity and everyday life. Adequate device programming along with ET could have a synergic action in improving functional capacity.

#### Implantable Cardioverter Defibrillator

Substantial evidence demonstrates that ET improves functional capacity without increased risk of shocks (83–85). During the HF-ACTION trial, only one subject experienced an ICD discharge that prevented at least a single supervised ET session from reaching the target duration or intensity (21).

According to a 2019 Cochrane Review, there were no differences in the risk of discharging appropriate shock, inappropriate shock, or all shock between the exercise-based CR group and the control group, neither at the end of intervention nor at a longer follow-up after the intervention period (84).

Living with an ICD can lead to anxiety and depression, and it often leads to social isolation and avoidance of physical activity. Furthermore, patients with ICD may reduce they participation to ET because of fear of shock. A systematic review concluded that −20% of ICD patients have clinically significant psychological distress (86). Psychosocial intervention could improve clinical and mental status of ICD recipients attending a CR program.

The COPE-ICD was a randomized trial designed to assess the effect of a comprehensive CR intervention, including ET and psycho-education in patients treated with an ICD (87).

The trial demonstrated that combining ET and a psycho-educational intervention could improve mental health besides the aerobic capacity.

Specific precautions should be observed in ICD recipients undergoing ET (83). ET programs should always be preceded by a maximal or symptom-limited exercise test. The electrophysiologist should optimize the “Pain Free” algorithms that should be programed to start at least 20 bpm over the maximal pulse rate; ICD shock threshold should be at least 30 bpm over patients' max HR.

During ET, traumatic damage to ICD should be avoided. Patients should start to train in a controlled environment; electrocardiograms should be monitored during the first sessions and every time exercise intensity is increased. Stable patients may later continue exercise in a non-supervised setting. Prevention of inappropriate shocks requires training at 10–20 bpm below the programed HR of ICD intervention.

#### Cardiac Resynchronization Therapy

CRT improves exercise capacity, QoL, and survival in patients with HF.

The addition of ET to CRT leads to further improvements in exercise capacity, hemodynamic measures, and QoL (88–90).

In CHF patients, an ET program should be prescribed after implantation in order to maximize the expected benefits and to obtain the best possible outcome from CRT.

A preliminary exercise test in CRT recipients is crucial to detect a loss of resynchronization during physical activity. The disappearance of biventricular capture may be caused by a loss of atrial sensing, frequent premature ventricular contractions, atrial arrhythmias, and a spontaneous AV conduction, becoming shorter than the programed AV delay. Optimization of device setting reduces the risk of loss of resynchronization during exercise.

### Left Ventricular Assist Device Recipients

Therapy of HFrEF patients begins with modification of the patients' lifestyle and pharmacotherapy in order to prevent a progression of the disease and to maintain the heart's function (64). If these approaches do not succeed, heart transplantation (HTx) is considered the therapeutic gold standard. For suitable patients, HTx continues to be limited by donor shortages all over the world, despite focused medical, social, and governmental efforts to increase awareness and donation rates (64, 91). With persistently high rates of waiting list and deaths for those on the HTx list, despite implantable defibrillators, cardiac resynchronization, and optimal medical therapy, a therapeutic alternative is represented by LVAD implantation.

The US Food and Drug Administration approved the first LVAD for treatment of advanced HFrEF 30 years ago. An LVAD is a mechanical device that is used to circulate blood; the inflow cannula is attached to the LV apex, and blood is pumped into the ascending aorta. A percutaneous driveline connects the pump to an external controller, powered by two batteries. The controller regulates pump function and provides digital messages and alarms.

Over the last few years, tremendous progress has been made with the newer LVADs. Modern devices are small and function almost silently: LVAD is more reliable, and its durability has increased, whereas, device-related complications have drastically decreased (but not eliminated) as compared with earlier generations of devices.

Despite the lack of generally accepted recommendations for LVAD recipients, evidence is accumulating that CR is beneficial. The goal is to return recipients to a normal and independent life. Besides the typical goals of CR, which include improvements in functional capacity and strength, specific additional goals in LVAD patients are extended to education and understanding the operation and handling of the device, self-management of anticoagulation, and psychological and social counseling. In addition to the CR immediately after LVAD implantation, repeated CR can be necessary in patients who exhibit adverse events (e.g., neurological complications) or those presenting with extreme deconditioning.

All LVAD recipients should undergo exercise-based CR, as they have persistent functional impairment related to peripheral mechanisms such as skeletal muscle deconditioning and apoptosis, derangement of skeletal muscle structure, and metabolic function that are associated with exercise intolerance. In addition, recruitment of skeletal muscle fibers during exercise increases oxygen transport and uptake by skeletal muscles and upregulation of anabolic enzymes, decreasing the catabolic catalysts. These favorable changes lead to an increase in peak muscle strength (92).

Patients with neurological complications should undergo rehabilitation in a center with combined cardiac and neurological rehabilitation facilities. For the patient with complex neurological problems, the intervention goals should be modified. Some conditions may significantly limit the patient's functional mobility. These conditions include (1) altered mental status with inability to follow commands and minimal ability to participate in planned activities; (2) cerebral vascular accident with significant loss of muscle function and inability to bear weight; (3) cardiovascular instability on high-dose or multiple inotropic drugs; and (4) significant impairment of oxygenation that requires high oxygen concentration, paralytic drugs, or sedation.

In these situations, the physical therapist continues to work to achieve the highest level of function possible within medical limitations. As the medical condition improves and the recipient is able to participate in planned activities, the physical intervention program is advanced to focus on EM and ambulation, as tolerated.

In every patient, as well as in LVAD recipients, EM is the first step for initiation of exercise therapy (93–95).

Before EM and ET programs, medical history and clinical and functional evaluations are requested. Vital signs, self-reported symptom scores, and LVAD function should be monitored (Table 2). The goals of EM can be summarized as follows: (1) to prevent postoperative complications due to bed rest; (2) to minimize loss of mobility; (3) to maximize independence; and (4) to facilitate weaning from the ventilator (93–95).

**Table 2:** Instruction to reduce the risk of adverse events when early mobilization starts in LVAD patients.

•Assessment *(persistence of VAD-related and HF symptoms, medications and particular treatments, i.e., need for continuous or intermittent infusions, ventilator settings or oxygen requirements prescribed)*
•Recent and past medical history, and level of exercise capacity previous to disease state.
•Mental status and cognitive ability.
•Vital signs and risk of cardiovascular instability.
•Screen range of motion, coordination, balance, strength, endurance, functional capacity.
•Haemochromocytometric, ionic and renal functional assessment: *start EM and/or ET when hemoglobin >9 g/dl, sodium >130 mEq/L, potassium >3.8 mEq/L, and/or creatininaemia <1.9 mg/dl*.
•Follow sternotomy and skin integrity.
•Patients should always wear a driveline stabilization belt during EM and/or ET.
•The patient should have his/her travel bag nearby at all times.
**Promote:**
1. Low-to-moderate intensity dynamic large muscle group work.
2. Walk & talk' approach is suggested.
3. Organize an appropriate place to put monitor, console-controller and batteries; the VAD equipment location should not impede emergency procedures during ET.

*LVAD, left ventricular assist device; HF, heart failure; EM, early mobilization; ET, exercise training*.

The preliminary phase is not standardized, but it is conditioned by the patient's clinical status, facilities, and timing of referral. EM resembles passive and active motions. Once the patient is out of bed, leg raising and hip girdle mobilizations are useful as a preparation to transfer weight from sitting to standing; then ambulation should be initiated, initially in the patient's room, progressing later to the ward. Later on, a set routine consisting of bicycle, treadmill, and upper body ergometer is followed (Table 3). The frequency of EM sessions is once a day, 6–7 days per week, with a duration varying from 15 min to 1 h, as tolerated (94). According to the patient's clinical needs, EM should be adapted (93).

**Table 3:** Early mobilization and exercise modalities in e-CR: step-up mobilization (*duration of single exercise is not contemplated and neither is step-back if clinical problems occur*).

1. Positioning and bed mobility activities; sitting on edge of bed. Move all extremities in sitting position; Move all extremities in sitting position.
2. Sit in chair.
3. Move all extremities in standing position. Ambulate with assistance: training is allowed with rolling walker.
4. Breathlessness management and recovery strategies.
5. Attempt to achieve a target of 11 to 14 out of 20 of the Rate of Perceived Exertion scale.
6. Patient's native heart rate should not exceed 120 beats/min during exercise.
7. Full range of motion.
8. Progressive ambulation.
9. Walk on level surface.
10. Treadmill walking.
11. Independent ambulation.
12. Begin stair climbing.
13. Lifting small weights.

*CR, cardiac rehabilitation*.

A flexible approach ensures safe EM. Attention should be directed to avoid abrupt postural changes and body balance issues that may result from the equipment bag, weighing from 2 to 2.5 kg. Unintentional disconnection from the VAD external power supply has been described (96). As regards the timing to start, EM should be contemplated as soon as the patient's hemodynamic and clinical status is stable and LVAD is correctly working.

No guidelines describing the specific ET setting, modality, and duration for LVAD-supported patients are available; only limited evidence of implementation of light exercise intensities is accessible (96). Although, it is reasonable to assume that longer ET interventions could improve functional capacity and QoL, long-term adherence to these interventions has not yet been described. ET should be performed using bicycle ergometer to minimize the risk of falls (96) and guided by the perceived level of exertion as measured by the modified Borg scale. Alternatively, a CPET can be used to guide ET to optimize exercise workload prescription (32). This may permit a “threshold-based” aerobic exercise intensity prescription (32). Additionally, some exercise activities may exert torsion on the driveline and, therefore, must be avoided; running, rowing machine, cross trainer, abdominal exercises, bilateral arms above the head with weights, or abduction with weights or swimming is contraindicated (96).

ET may need to be modified in the following situations: infections, arrhythmias orthostatic hypotension, and any new alarms or VAD malfunction, during exercise sessions. Limited evidences of implementation of light exercise intensities are available (97–109). In the largest experience, although, CR was recommended, 348 (30%) of Medicare beneficiaries participated in CR. CR was associated with a 23% lower 1-year hospitalization risk and a 47% lower 1-year mortality risk (110).

LVAD patients undergoing supervised ET demonstrated significant improvement in exercise capacity and QoL scores when compared with the usual care group, with no serious adverse events with exercise. These results suggest that supervised ET is safe and can improve outcomes in LVAD patients (111).

The impact of device therapy continues to increase and permeate the medical community: it is imperative to disseminate knowledge and experiences. LVAD recipients show a significant exercise improvement after implantation, but their functional capacity remains sub-optimal; despite LV unloading, functional capacity is below 50% of predicted VO_2peak_. ET might provide additional benefit, but there is little evidence. There is room for improvement, and there is an astounding opportunity for CR to promote and standardize specific exercise regimens. The challenge that lies ahead is formidable.

## Conclusion

In spite of some open questions, there is a consensus about the benefit of CR for patients with HFrEF. ESC guidelines for HF treatment recommend aerobic exercise as a class I treatment to improve functional capacity and to reduce the risk of hospitalization and death (64). American Heart Association/American College of Cardiology (AHA/ACC) guidelines confer to CR a class I recommendation for patients with HF to improve functional status and a class IIa recommendation to improve functional capacity, exercise duration, and health-related QoL and to reduce mortality (91).

The British National Institute for Health and Care Excellence (NICE) recommends people with HF a personalized, exercise-based CR program, provided in a format and setting easily accessible for the patient (112).

The relevance of introducing into the CR programs clinical components, such as etiological assessment, risk stratification, optimization of medical therapy, and management of comorbidities, highlights the value of CR as an integrated component of the management programs for patients with HF (64).

Most of our current knowledge in the field derives from studies performed on stable middle-aged patients with HFrEF, but encouraging results have been observed when CR was offered to other phenotypes of HF and in other phases of the disease trajectory.

A personalized approach by optimization and tailoring the rehabilitative program according to patient's characteristics is the probable future scenario for advances in this field. Financial feasibility, underuse, and poor adherence represent issues that should be faced to deploy the maximum potential of CR.

Telemedicine with its many possible applications for patients with HF may increase participation and overcome some of the barriers that limit CR utilization.

## Author Contributions

AP, ML, and DS contributed to conception and design of the review. AP, UC, MP, SS, LADV, FG, and MB wrote sections of the manuscript. All authors contributed to manuscript revision and read and approved the submitted version.

## Conflict of Interest

The authors declare that the research was conducted in the absence of any commercial or financial relationships that could be construed as a potential conflict of interest.

## Publisher's Note

All claims expressed in this article are solely those of the authors and do not necessarily represent those of their affiliated organizations, or those of the publisher, the editors and the reviewers. Any product that may be evaluated in this article, or claim that may be made by its manufacturer, is not guaranteed or endorsed by the publisher.
